# The terminal region of the *E. coli *chromosome localises at the periphery of the nucleoid

**DOI:** 10.1186/1471-2180-11-28

**Published:** 2011-02-02

**Authors:** Jean-Christophe Meile, Romain Mercier, Mathieu Stouf, Carine Pages, Jean-Yves Bouet, François Cornet

**Affiliations:** 1 Université de Toulouse; Université Paul Sabatier; Laboratoire de Microbiologie et Génétique Moléculaires; F-31000 Toulouse; France; 2 Centre National de la Recherche Scientifique; LMGM; F-31000 Toulouse; France; 3 Current Address: Institute for Cell and Molecular Biosciences, The Medical School, University of Newcastle, 2nd Floor, Catherine Cookson Building, Framlington Place, Newcastle, NE2 4HH, UK

## Abstract

**Background:**

Bacterial chromosomes are organised into a compact and dynamic structures termed nucleoids. Cytological studies in model rod-shaped bacteria show that the different regions of the chromosome display distinct and specific sub-cellular positioning and choreographies during the course of the cell cycle. The localisation of chromosome loci along the length of the cell has been described. However, positioning of loci across the width of the cell has not been determined.

**Results:**

Here, we show that it is possible to assess the mean positioning of chromosomal loci across the width of the cell using two-dimension images from wide-field fluorescence microscopy. Observed apparent distributions of fluorescent-tagged loci of the *E. coli *chromosome along the cell diameter were compared with simulated distributions calculated using a range of cell width positioning models. Using this method, we detected the migration of chromosome loci towards the cell periphery induced by production of the bacteriophage T4 Ndd protein. In the absence of Ndd production, loci outside the replication terminus were located either randomly along the nucleoid width or towards the cell centre whereas loci inside the replication terminus were located at the periphery of the nucleoid in contrast to other loci.

**Conclusions:**

Our approach allows to reliably observing the positioning of chromosome loci along the width of *E. coli *cells. The terminal region of the chromosome is preferentially located at the periphery of the nucleoid consistent with its specific roles in chromosome organisation and dynamics.

## Background

Bacterial genomes appear as compact DNA masses, termed nucleoids, located centrally along both the length and width of the cells [1]. Nucleoids are highly organised structures within which each chromosome region occupies specific locations along the length of the cell and displays a distinct choreography during the cell cycle (for reviews: [2,3]). In most bacteria, nucleoids contain a single chromosome replicated from a single origin. This defines two oppositely oriented replichores, each extending from the replication origin, *oriC *to the terminal (*ter*) region, oppositely located on circular chromosomes. This replicative organisation has important consequences for the global organisation and segregation of bacterial nucleoids. In *E. coli*, replication occurs around the cell centre (i.e., the mid-cell position) [4]. Segregation is concomitant with replication so that replicated loci are segregated from mid-cell to the equivalent positions in the future daughter cells (the quarter positions) following the order of their replication [5-9]. The *oriC *region (*ori*) is thus the first to segregate, and the *ter *region the last. In newborn cells, loci of the *ter *region are located close to the new cell pole (polar positioning) and migrate towards the midcell during the replication process.

Recent advances in bacterial cell cytology allow a general model of the *E. coli *nucleoid structure to be established. The *ori *region, located towards midcell, migrates to the quarter positions after being duplicated. The two replichores occupy distinct locations on each side of *ori *with chromosome loci recapitulating the *ori-ter *genetic map along the cell length axis [7,10,11]. In this model, the *ter *region is inferred to contain a stretched region linking the two nucleoid edges [12,13]. This linking region is believed to be composed of a segment of 50 kb randomly taken within the 400 kb *ter *region. Notably, the *ter *region is the site of specific activities involved in segregation [14,15]: in particular, it interacts with the MatP protein [16] and with the FtsK DNA translocase ([17]; our unpublished results).

In addition to this replichore organisation, the *E. coli *nucleoid appears to be structured into macrodomains (MDs). MDs are 0.5 to 1 Mb regions inferred to be self-compacted and composed of loci having similar intracellular positioning and dynamics during segregation [6,9,18]. The *E. coli *chromosome contains four MDs: the Ori and Ter MDs (containing *ori *and *ter*, respectively) and the Right and Left MDs flanking the Ter MD. The two regions flanking the Ori MD, called the non-structured regions (NS regions), do not display MD properties and contain loci displaying a higher intracellular mobility than MD-borne loci [9].

Most studies of the localization of chromosomal loci in bacteria have focused on their position along the length of the cell. We are not aware of any reported data concerning loci positioning across the width of the cell; this is partly because bacteria are too thin for accurate 3-D analysis, by for example confocal microscopy. We evaluated the position of *E. coli *chromosomal loci across the width of cells from statistical analysis of 2-D images. We observed the distributions of loci tagged with fluorescent proteins and compared them to simulated distributions from different cell width positioning models. Using this method, we detected different positioning patterns for different loci across the cell width. Loci in the *ori *region and Right MD appeared to position randomly across the nucleoid width. A locus in the NS-right region was preferentially located close to the cell centre, whereas a *ter*-borne loci localised at the nucleoid periphery. To validate these observations, we demonstrated that our method reliably detects the migration of individual loci, as part of the global migration of the nucleoid towards the cell periphery induced by production of the bacteriophage T4 Ndd protein.

## Results

### Positioning of chromosome loci in living cells

**Figure 1:**
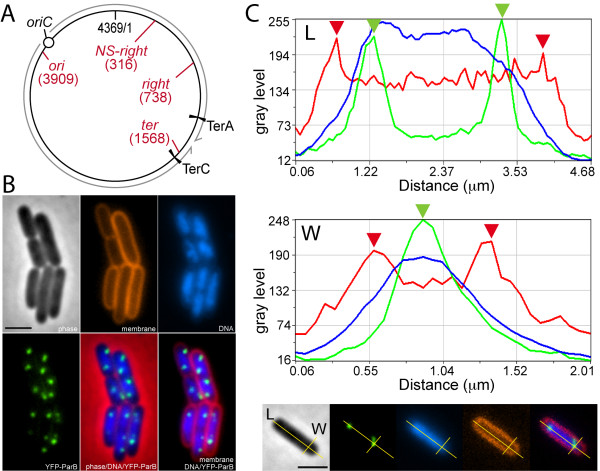
**Positioning of chromosome loci in living cells**. (A) A scheme of the *E. coli *chromosome with relevant features indicated. The replication origin (*ori*) and the two inner replication terminators (TerA and TerC) defining the zone of replication termination are shown. The grey arrows indicate the sense of replication. The loci used for insertion of the *parS *site are shown in red. Coordinates are in kb. (B) Micrographs of cells harbouring the YFP-ParB foci at the *ori *locus. From top left to bottom right: phase contrast; membrane staining (FM 4-64); DNA staining (DAPI); YFP-ParB foci; overlay phase/DNA/YFP-ParB; overlay membrane/DNA/ParB. (C) Linescan analysis of fluorescence signals along cell length (L, top panel) and cell diameter (W, middle panel). Linescans of fluorescence intensities (Y-axis, in Gray Level units) for the cell membrane (red); DNA (blue) and YFP-ParB (green) are shown along the two cell axes (X-axis in μm). Red arrowheads indicate the cell boundaries and green arrowheads show the positions of YFP-ParB foci. The bottom panel shows micrographs of the cell scanned in the panels above with the two linescans used (from left to right: phase contrast; YFP-ParB; DNA; membrane; overlay YFP-ParB/DNA/membrane). Scale bars are 2 μm.

To label chromosomal *loci *such that their position could be determined, we used insertions of the *parS *site from the bacteriophage P1 and production of the YFP-Δ30ParB fusion protein (Methods) [19,20]). The *parS *site was first inserted at four different loci located at 3909 kb (*ori*), 316 kb (*right*, inside the right MD), 738 kb (*NS-right*) and 1568 kb (*ter*) on the *E. coli *chromosome map (Figure 1A). The resulting strains showed equivalent growth rates and normal cell shape whether or not they produced the YFP-Δ30ParB protein (doubling times in synthetic medium of 45 min. at 42°C and 70 min at 30°C).

**Figure 2:**
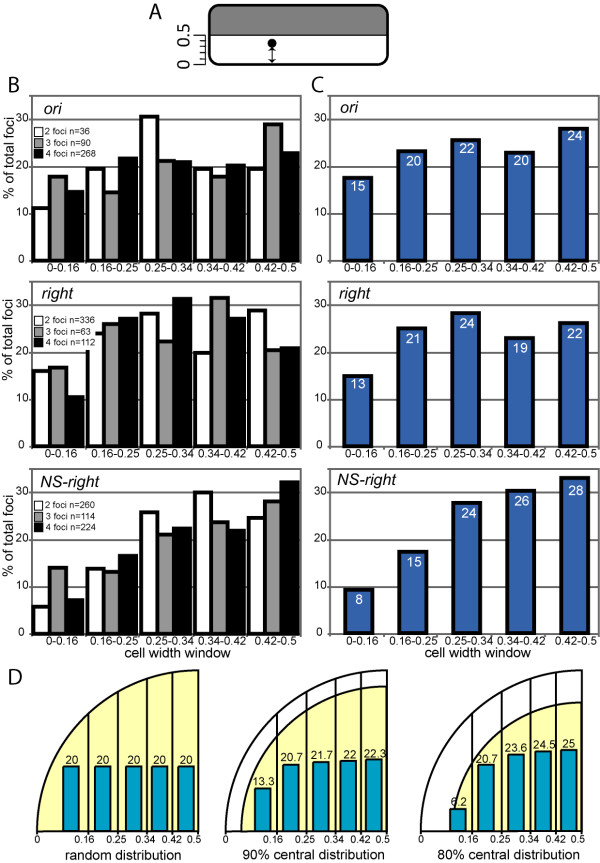
**Distributions of foci along the cell diameter**. (A) Drawing showing the measurement of the apparent positions of foci along the cell diameter. Distances along the cell diameter between the centres of foci and the nearest membrane were measured. (B) Distributions of foci along the cell diameter for the *ori, right *and *NS-rigth *loci in the various cell classes. Distributions are plotted as the percentage of total foci in each cell class (Y-axis). The sample size of the cell classes is given on each graph. The positions of foci were subdivided into five windows corresponding to cell slices of equivalent areas (from 0 at the cell periphery to 0.5 at the cell centre, X-axis). (C) Data from (B) were compiled into single distributions. The percentage of foci in each cell width window is given on the histograms. (D) Examples of simulated distributions. A quarter of a cell section is shown with five cell slices (X-axis). Yellow areas show areas of permitted localisation of foci for each model. The corresponding distributions of foci in the five cell width slices are shown as histograms with the corresponding percentage of total foci.

Transient production of the YFP-Δ30ParB protein allowed the visualisation of fluorescent foci in most cells (> 90%, Figure 1B and data not shown). Membrane and DNA dyes were used concomitantly to visualise the cell periphery and the nucleoid (Figure 1B and 1C). Cells were classified into populations defined according to their number of foci, and the positioning of foci along the length of cells was evaluated for each population (Figures 1C and 2). The distances of the foci to the closest cell pole were scored on a five points scale along the long axis of the cell from the pole to mid-cell (Additional file 1, Figure S1). The *ori*, *right *and *NS-right *loci displayed 2 to 4 foci that mostly found at or near the quarter positions, whereas the *ter *locus displayed 1 or 2 foci, which were mostly located at mid-cell (Additional file 1, Figure S1). The proportion of mid-cell-located *ter *foci was lower for cells harbouring a single focus than for cells with two foci, consistent with a progressive migration of the *ter *region from the new cell pole to the mid-cell during the cell cycle [7,8,21]. These findings are consistent with previous observations using similar [9,20] or different detection systems and growth conditions [6,10].

### Positioning of chromosome loci along the cell diameter

**Figure 3:**
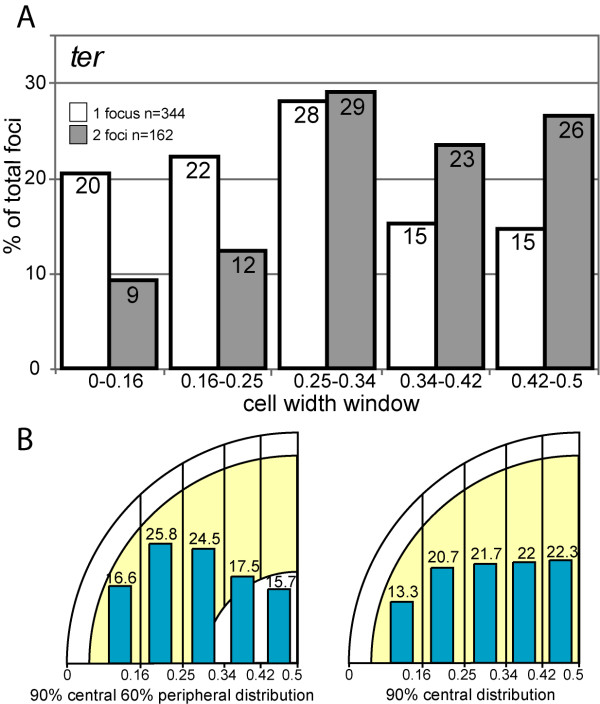
**Distribution of *ter *locus foci along the cell diameter**. (A) Distributions of foci along the cell diameter for the *ter *locus in the two cell classes. Legend as for Figure 2B. (B) Examples of simulated distributions. Legend as for Figure 2D.

**Figure 4:**
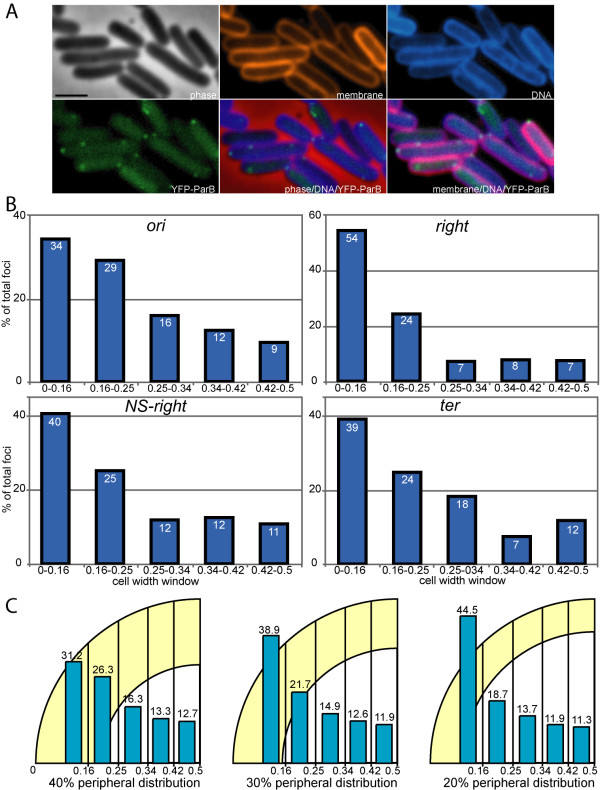
**Distributions of foci along the cell diameter in Ndd-treated cells**. (A) Micrographs of Ndd-treated cells showing the relocation of chromosomal DNA towards the cell periphery. Legend as for Figure 1B (*parS *site inserted at the *ori *locus). (B) Distributions of foci of the indicated loci along the cell diameter. Legend as for Figure 2C. (C) Legend as for Figure 2D.

The position of a fluorescent focus along the width of the cell cannot be directly determined using 2-D wide-field microscopy. Indeed, a focus located near the cell periphery may appear at the centre of the cell diameter or at the edge according to the orientation of the cell cylinder with respect to the focal plan. Nevertheless, since the orientations of the cell cylinder are expected to be random for a population of rod-shaped bacteria deposited on a plane surface, the mean position of particular foci can be calculated from the apparent distributions of foci along the cell diameter. We therefore measured the apparent distance along the cell diameter between foci and the membrane (Figure 1C). The datasets obtained were then compared with distributions calculated for different models of positioning across the width of the cell (Methods). We defined five slices of equivalent surface in a quarter of the cell section and calculated the expected distributions of foci according to the various models of positioning (the 2-D apparent foci distributions for various 3-D localisation patterns are shown in Figures 2, 3 and 4).

Again, cells were classified into major populations depending on the number of foci they contained. For *ori*, *right *and *NS-right *loci, the distributions of foci did not differ significantly between cell populations. Thus, there was no obvious correlation between positioning and cell cycle progression (Figure 2B and data not shown). We therefore combined the datasets of the different classes into a single distribution (Figure 2C). The *ori *and right *loci *appeared to be similarly distributed into four axial sections, but were less frequently found in the most peripheral section (Figure 2C). Comparison of the observed and expected datasets using the χ2 test showed that the distribution of the *ori *and *right *loci was significantly different from all simulated distributions except the 90% central model (Figure 2D; χ2 = 2.7 and 2.8, respectively; corresponding to p-values of 0.6). The 90% central model is consistent with the mean position of the nucleoid, which appears as a central DNA mass partly excluded from the extreme periphery of the cell (Figure 1B). The *ori *and *right loci *thus appeared randomly positioned across the width of the nucleoid. The *NS-right *locus clearly tended to localise closer to the cell centre than the *ori *and *right *loci without being completely excluded from the cell periphery. However, we failed to find a model that corresponded to this distribution, the best p-value value obtained being 0.003 with the 80% central model (not shown).

In the case of the *ter *locus, only cell populations harbouring one or two foci were statistically relevant. In both populations, a large fraction of foci were located close to either the cell pole or the mid-cell position where the division septum forms (Additional file 1, Figure S1). We thus excluded cells with an apparent constricting division septum from our analysis to avoid variations due to local deformation of the membrane. In contrast to other loci, the distribution of *ter *foci clearly differed between the two cell populations (p-value < 10^-3^; Figure 3). The distribution of foci in cells with a single focus appeared more peripheral than random. Indeed, the distribution was significantly different from the random and central models (p-value < 10^-3^); the best fitting model was the 90% central 60% peripheral model in which foci are excluded from the 10% cell periphery and 40% cell centre regions (p-value = 0.1; Figure 3). Cells with two foci showed a distribution more central than random. It was however different from any simulated distribution (p-value < 0.05). This more central location is not due to local deformation of the membrane during constriction of the division septum since cells with a constricting septum were omitted from our analysis. The *ter *region is the last to be segregated, and consequently nucleoid segregation is almost completed when *ter *foci are duplicated [8]. It follows that duplicated *ter *foci located close to midcell lie at the mid-cell edge of the nucleoid. The distributions of foci of the *ter *locus in cells harbouring one or two foci thus indicates that the *ter *region is preferentially located at the periphery of the nucleoid, either close to the parietal membrane (in single foci cells) or close to a cell pole (after *ter *duplication) throughout cell cycle progression.

To rule out a specific behaviour of the *ter *locus used, we analysed a second *ter *locus located at 1490 kb (*trg*). The results reported in Additional file1 Figure S5 clearly show that the *trg *locus also preferentially localises at the nucleoid periphery in the cell population harbouring a single fluorescent focus. This strongly suggests that the peripheral location is a general property of the terminal region of the chromosome.

### Loci positioning after nucleoid disruption

We tested whether the same approach could detect a change in chromosome organisation. We used production of the Ndd (Nucleoid Disruption Determinant) protein from the T4 bacteriophage. Ndd disrupts the central and compacted structure of the nucleoid in *E. coli *and causes chromosomal DNA to delocalise to the cell periphery [22-24]. A plasmid carrying a T7p-*ndd2*^*Ts *^fusion was transferred into the strains carrying *parS *insertions, which express the T7 RNA polymerase (Methods). Strains containing the pT7-*ndd2*^*Ts *^plasmid had a doubling time similar to the parental strains in the absence of Ndd production (45 min. at 42°C in M9 medium). Ndd2^Ts ^production was induced by a rapid temperature shift down to 30°C in the presence of IPTG (Methods). Ndd2^Ts^-producing cells (hereafter called Ndd-treated cells) stopped dividing almost immediately and did not elongate more than 1 μm (not shown; [25]). The DNA was stained with DAPI and the cells examined by microscopy. Nucleoid disruption was observed after 15 min of induction and appeared to be complete in virtually all cells after 30 min. At this stage, the DAPI staining pattern was similar to the shape of the membrane, indicated that most of the cellular DNA was delocalised towards the cell periphery (Figure 4A and Additional file 1, Figure S2).

The number of foci per cell was lower in Ndd-treated than control cultures (Additional file 1, Figure S3). This suggests that Ndd prevents segregation of loci (see discussion). Fluorescent foci were nevertheless observed in most Ndd-treated cells and their size was indistinguishable from that of foci observed in control cells (Additional file 1, Figure S2 and data not shown), suggesting that Ndd does not affect the local structure or compaction of the DNA (see discussion).

We analysed the distribution of foci along the length of Ndd-treated cells (Additional file 1, Figure S4C). The *ori*, *right *and *NS-right *loci were more widely distributed in Ndd-treated than control cells and positioning at the quarter positions was lost or less accurate. A significant proportion of foci were close to the cell poles, consistent with migration of the DNA towards the periphery of the cell (Additional file 1, compare Figures S4C with S1). In contrast, the positioning of the *ter *locus was only slightly affected by Ndd (Additional file 1, Figure S4C): the pattern was generally unchanged although Ndd treatment was associated with mid-cell-located foci being frequent in both cell classes (1 and 2 foci) and pole-located foci more frequent in cells harbouring a single focus.

We next observed the distribution of foci along the cell diameter. We first analysed the cell classes independently and found no significant difference between their foci distribution (Additional file 1, Figure S4D). We thus used the total cell population as a single group for the subsequent analysis (Figure 4B). The distributions of the four loci along the cell diameter in Ndd-treated cells was very different from that in control cells (Figure 4): in Ndd-treated cells all loci appeared shifted towards the cell periphery (Figure 4B). Comparison with simulated distributions showed that the observed distributions were consistent with the loci being excluded from the 60 to 80% centre part of the cell width (Figure 4C and not shown; p-values were lower than 0.05 with all models except the 20 to 40% peripheral models). We conclude that our analysis can detect modifications of the positioning of chromosome loci across the width of the cell, and this strengthens the validity of our findings concerning positioning in the absence of Ndd production.

### Correlation between loci positioning along cell length and width

**Figure 5:**
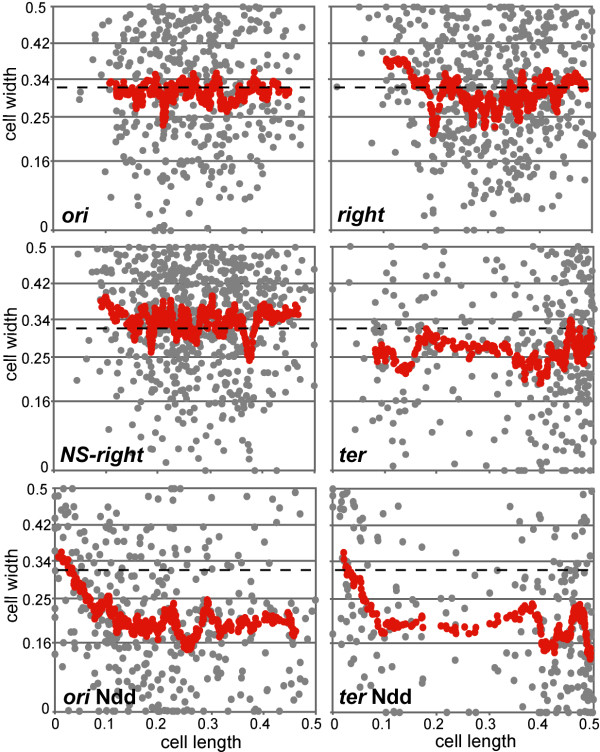
**Analysis of correlation of the position of foci along the cell length with that along the cell diameter**. Graphs show the positions of foci of four loci in wt and Ndd-treated cells, as indicated in each panel, along the cell diameter (Y-axis) as a function of their position along the cell length (X-axis). The grey dots are individual foci. The red dots are sliding means of twenty adjacent foci (with a step of one focus). For the *ori*, *right *and *NS-right *loci in Ndd-untreated cells and for the *ori *and *ter *loci in Ndd-treated cells, the data from the different cell classes were combined, as these dataset do not statistically differ (see Figure 2). In the case of the *ter *locus in Ndd-untreated cells, only the data from cells with a single focus are plotted. The dotted lines show the mean position of foci calculated from the 90% central model.

Foci were sorted in ascending order of their distance to the closest pole (X-axis) and their position along the cell diameter was plotted (Y-axis, grey dots; Figure 5). No correlation appeared for any locus and calculated Pearson correlation coefficients were not significant (less than 0.05 in absolute value). The datasets of cell diameter position are *de facto *highly variable. Therefore, sliding means for 20 adjacent dots were calculated and plotted to help visualise patterns (red dots, Figure 5). Again no general relationship between position along one axis and position along the other could be established. Nevertheless the *ori *and *right *loci appeared to behave similarly and the *NS-right *locus tended to be closer than *ori *and *right *to the cell centre. The *ter *locus was more peripheral than other loci in cells with a single focus (red dots). The same analysis was performed for the *ori *and *ter *loci after Ndd treatment (Figure 5). For the *ter *locus, distributions of the two cell classes were combined since they were not significantly different (Additional file 1, Figure S4D). In both cases, the sliding mean was consistent with the peripheral location of the loci. Equivalent patterns were obtained for the *right *and *NS-right *loci in Ndd-treated cells (not shown). Foci located in the 0-0.1 cell length slice were more central than the other foci. This cell length slice corresponds to the cell poles, where the membrane curvature modifies the cell width distribution of foci. This effect was detected only in Ndd-treated cells due to the enrichment of loci in this cell slice compared to control cells (Additional file 1, Figure S4C).

## Discussion

We report that it is possible to assess the mean position of chromosome loci across the width of a rod-shaped bacterium using two-dimensional pictures. We recorded the apparent position of fluorescence-tagged chromosomal loci along the diameter of a large number of cells and compared the resulting distributions to simulated distributions calculated from different positioning models. We analysed five loci mapping in four different chromosomal regions that behave differently during the cell cycle. For these five loci, we detected three different patterns, showing that our method can detect differences in cell width localisation. The *ori *and *right *loci appeared randomly distributed through a cell volume corresponding to the nucleoid, whereas the *NS-right *locus was more central and *ter *loci more peripheral. Our method based on the analysis of individual loci successfully detected the general migration of chromosomal DNA towards the cell periphery provoked by the production of the Ndd protein. Indeed, in Ndd-producing cells, the four loci assayed were clearly distributed at the cell periphery. This observation validates the differences observed in the localisation of these loci in normal cells. This is, to our knowledge, the first successful attempt to localise the position of chromosome loci along the short axis of bacteria.

The method used here involves assessing mean distributions such that general tendencies of positioning across the cells can be assessed, rather than rapid or transient changes in position. Indeed, the possible movements of loci during replication, subsequent segregation or gene expression are likely to be too fast to affect significantly the distributions observed in this way. Loci may thus have transient preferential cell width localisations, for instance at the cell periphery during segregation of newly replicated DNA [26] or during gene expression [27,28], that our method would fail to detect.

The emerging view of the large-scale organisation of the *E. coli *nucleoid along the long axis of the cell is that it is organised from the *ori *region, with the left and right replichores recapitulating the genetic map on each side of *ori *and the *ter *region forming a less condensed region linking the two edges of the nucleoid [12,13]. The chromosome also contains four macrodomains: Ori, Right, Left and Ter, that occupy distinct chromosome territories and two less structured regions (NS-right and left) that are less accurately positioned [9]. Our results have implications both the global replichore organisation and the macrodomain organisation of the chromosome. Loci located in the Ori and Right macrodomains (the *ori *and *right *loci) conformed to a random localisation model in the nucleoid width, suggesting that macrodomains do not occupy specific locations in the cell diameter. Thus, macrodomain territories only concern nucleoid length and not nucleoid layers along the width of the cell. The *NS-right *locus behaves differently from the macrodomain loci, suggesting that the different features of macrodomain and NS regions involve a different positioning along cell width. The more central than random localisation of the *NS-right *locus may appear contradictory with the higher mobility described for this chromosome region [9]. We would stress however that there is no obvious direct link between the mobility and the mean positioning of a chromosome locus. The NS-right locus may still move faster but in a more confined region in the cell width compared to loci located in macrodomains. The *ter *loci shown a particular localisation in cells with a single focus: they were more peripheral than other loci. Comparison with simulated models indicates that these loci are excluded from the cell centre. This peripheral location of *ter *is not restricted to any particular step of chromosome dynamics since it appears conserved for foci located from the pole to the middle of the cell (Figure 5 and Additional file 1, Figure S5). The *ter *region migrates from the new cell pole to the mid-cell position during chromosome replication [8,21]. This movement along the cell length occurs before *ter *replication (i.e., in cells with a single *ter *focus). Our results strongly support the view that the *ter *region migrates from the cell poles to mid-cell along the periphery of the nucleoid. This is also fully consistent with the notion that at least a part of the *ter *region connects the nucleoid edges via a peripheral link [12,13]. It will be interesting to investigate if this particular behaviour of the *ter *region is related to specific features of this region such as the presence of *matP *sites [16] or the action of the FtsK translocase.

We used the T4 Ndd protein to interfere with chromosome organisation. Production of Ndd causes the centrally positioned nucleoid to move to the cell periphery by an unknown mechanism [24]. Following Ndd production and consequent nucleoid disruption, foci were detected as efficiently as in control cells (Figure 4A), indicating that the delocalised DNA remained fully proficient for ParB binding and spreading over *parS *sites. Moreover, ParB binding to *parS *requires IHF, and IHF-ParB complexes strongly prefer supercoiled substrates [29]. Therefore, effective foci visualisation in our experiments involving rapid Ndd action indicates that DNA supercoiling is not affected during Ndd-induced nucleoid delocalisation, consistent with previous observations during a slow Ndd disrupting process [24]. Ndd production reduced the number of foci per cell, particularly for the *ori*, *right *and *NS-right *loci (Additional file1, Figure S3). This effect was less pronounced for the *ter *locus indicating that it is not primarily due to a defect in the detection of foci. Following Ndd production, cell division is stopped more rapidly than chromosome replication [24], so the reduction in the number of foci per cell cannot be due to a reduction of locus copy number. The smaller number of foci number may in part be due to the peripheral location of the chromosome in Ndd-treated cells. Indeed, the thickness of the peripheral DNA, as measured by DAPI staining, appeared to be in the same range as the optical resolution limit (about 200 nm, i.e., 3 pixels; see Additional file 1, Figure S2). Therefore, foci in close proximity inside disrupted nucleoids would appear as a single signal. Thus, the apparent reduction in the number of foci per cell strongly suggests that segregated sister loci are brought back together during nucleoid disruption. Chromosomal loci are therefore not completely free as they relocate toward the membrane during nucleoid disruption but conserve some positioning information. Consistent with this, the delocalisation of foci along the length of the cell is not complete in Ndd-treated cells, at least for the *ori*, *right *and *ter *loci (Additional file 1, Figure S4C). Interestingly, the *ori *locus tends to localise close to the cell poles in cells with disrupted nucleoids, whereas the *right *and *ter *loci localise towards midcell. This suggests that Ndd action changes the intracellular orientation of the chromosome. We conclude that Ndd affects functions that maintain the central compaction and the orientation of the chromosome without provoking a complete disorganisation of the chromosomal DNA.

## Conclusions

We have developed an approach that allows to reliably observing the mean positioning of fluorescent objects along the width of rod-shaped bacterial cells from two-dimension images. We have successfully used this approach to study the positioning of *E. coli *chromosome loci and shown that loci of different chromosome region position differently along cell width. Most interestingly, loci of the terminal region of the chromosome are preferentially located at the periphery of the nucleoid consistent with the specific roles of this region in chromosome organisation and dynamics.

## Methods

### Strains and plasmids

Most strains used were derived from DLT812 (CB0129 Δ(*ara-leu*) *zac3051*::Tn*10 *[30]), rendered lysogen for λDE3 using the λDE3 lysogenisation kit (Novagen), and *pcp18*::*araE, FRT-Kn-FRT *by transduction to obtain DLT1886. The Kn resistance cassette was removed by transitory expression of Flp recombinase from pCP20 [31], yielding strain DLT1915. The *parS*-Kn cassette at positions 3909 kb (*ori*) and 1568 kb (*ter*), and the *parS-FRT-Cm-FRT *cassette at positions 316 kb (*NS-right*) and 738 kb (*right*) (see map Figure 1A) were transferred into DLT1915 from strains CC4711, CC4713 [19] and from strains carrying the NSR-3 and Right-3 [9] to yield strains FC542, FC543, FC541 and FC540, respectively. Insertion of the *parS-FRT-Cm-FRT *at the *trg *(1490 kb) locus of strain LN2666 (CB0129 *rpsL *(StR)) was obtained using standard transgenesis procedure with the λred system [19]. Transformation by pCP20 was used to remove the Cm resistance gene. To obtain the Ndd-producing plasmid pRM7, a fragment carrying *lacI *and a pT7-*ndd2ts *fusion [25] was ligated as a *Nru*I-*Hind*III fragment into pACYC184. Plasmid pBAD24-YFPΔ30ParB was used to produce the YFP-ParB fusion (gift from O. Espeli).

### Cell growth and microscopy

Strains carrying plasmids pBAD24-YFPΔ30ParB, and either pACYC184 (Ndd untreated cells), pRM7 (Ndd-treated cells) or no second plasmid (LN2666 derivative), were grown overnight at 42°C (derivatives of DLT1915) or 30°C (derivative of LN2666) in M9 medium supplemented with 0.2% casamino acids, 0.4% glucose; 2 μg/ml thiamine; 20 μg/ml leucine, 20 μg/ml thymine, 100 μg/ml ampicillin and, when required, 10 μg/ml chloramphenicol. These cultures were diluted 1/100 in the same medium and grown at the same temperature to an OD_600 _of 0.5-0.6. In the case of DLT1915 derivatives, the temperature was rapidly decreased by mixing the cultures with an equal volume of cold M9 medium, supplemented with 1 mM IPTG and 0.1% arabinose, followed by incubation at 30°C for 15 min. In the case of the LN2666 derivative, 0.1% arabinose was added to the culture followed by incubation at 30°C for 15 min. The dyes DAPI and FM4-64 were added to the culture to label DNA and cell membranes, respectively, and the cultures incubated for a further 15 min.. Aliquots of the culture were directly deposited on glass slides covered with a layer of 1% agarose containing M9 medium, and observed by phase-contrast and fluorescence microscopy using an inverted Olympus X81 microscope carrying a 100× oil-immersion Olympus lens (N.A. of 1.3) and a Roper CoolsnapHQ CCD camera. Images were acquired using Metamorph software.

### Measurement of foci position

Using Metamorph software, images of cell membranes, YFP-ParB signals, DNA and phase-contrast were artificially coloured in red, green and blue and merged. The Linescan function was used to analyze fluorescence signal intensities. Lines were drawn across the long and short axes of each cell and for each pixel of the lines, fluorescence intensities were measured for membrane (FM4-64, red), DNA (DAPI, blue) and YFP-ParB (green) signals. Data were plotted as intensity (grey level) *vs*. pixel distance along each line (Figure 1B). Along both axes, cell boundaries and the centre of YFP-ParB foci can be precisely determined as the positions of maximum intensity of the fluorescence signals (red and green arrowheads, respectively, in Figure 1B). Data were collected and calculated using Excel software. Apparent distances between the foci and the membrane were always measured to the closest pole (cell length) or parietal membrane (cell width) and the obtained values are reported as ratios relative the total cell length or diameter, respectively, such that the values are necessarily between 0 and 0.5. Cells were classified into populations according to the number of foci they contain. Cell length values were sampled into five cell slices of equal length. For cell diameter slices, we considered the *E. coli *cell to be a cylinder, and its transversal section a circle. The apparent distance of foci to the closest parietal membrane was then considered as its projection on the circle radius. The circle quarter was divided into five slices of equal area and the measured positions of foci along the transversal section were classified into theses slices. The measured cell diameter was 0.89 +/- 0.12 μm on average (428 cells), corresponding to slices ranging from 0.14 μm (for the most peripheral) to 0.07 μm (for the most central). If foci were randomly positioned along the cell width, they would be expected to be evenly distributed among the cell slices.

### Calculation of models and statistical analysis of datasets

To construct models of positioning across the width of the cell, we first reasoned that in the case of random positioning, the probability of finding a focus in a given cell slice is proportional only to the area of this slice (i.e., 20% in the case of five slices of equal areas). We then considered different theoretical distributions for foci between slices if excluded from increasing percentages (with 10% steps) of the cell periphery and/or the cell centre by subtracting circle areas (examples are shown in Figure 2, 3 and 4). Observed distributions were compared to calculated distributions using the χ2 test http://www.graphpad.com/quickcalcs. Distributions were considered to be different if the associated p-values were less than 0.05. Pearson's correlation coefficients between cell length and cell width distributions were calculated using Excel software.

## Authors' contributions

JCM performed most experiments (strain construction and microscopy), analysed the data and wrote an early version of the paper. RM performed early experiments (strain construction and microscopy) and analysed the data. MS performed the experiments in Additional file, Figure S5 (strain construction and microscopy). CP constructed some of the strains. JYB constructed some of the strains, designed, analysed and interpreted the experiments, and wrote the paper. FC designed, analysed and interpreted the experiments, and wrote the paper. All authors read and approved the final manuscript.

## Supplementary Material

Additional file 1 **Additional figures**. Figures S1, S2, S3, S4 and S5. Click here for file
