# Supplementary material for: The terminal region of the E. coli chromosome localises at the periphery of the nucleoid
Source: BMC Microbiol. 2011 Feb 2;11:28. doi: 10.1186/1471-2180-11-28 (PMC3040692; doi:10.1186/1471-2180-11-28)
Supplement: Additional file 1 — Additional figures. Figures S1, S2, S3, S4 and S5. [file 1471-2180-11-28-S1.PDF]

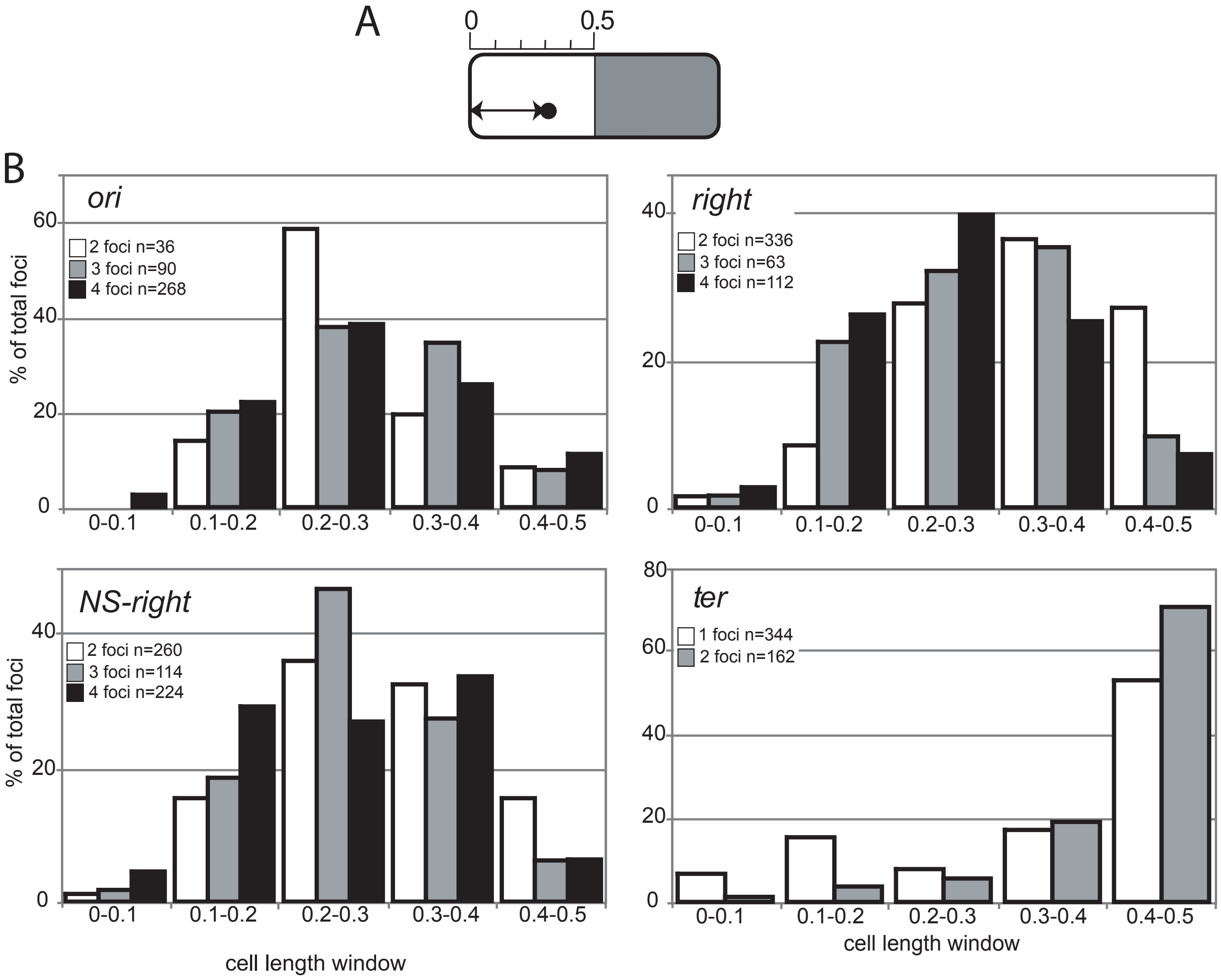

Figure S1: Positioning of foci along cell length.
(A) Drawing showing the measurement of the positions of foci along cell length. The distances of foci centres to their closest cell pole were measured. (B) Distributions of foci of the indicated loci along cell length in the different cell classes. Distributions are plotted as the percentage of total foci in each cell class (Y-axis). The sample size of the cell classes is given on each graph. Foci positions were sampled into five cell length windows (from 0 at the cell periphery to 0.5 at the cell centre, X-axis).

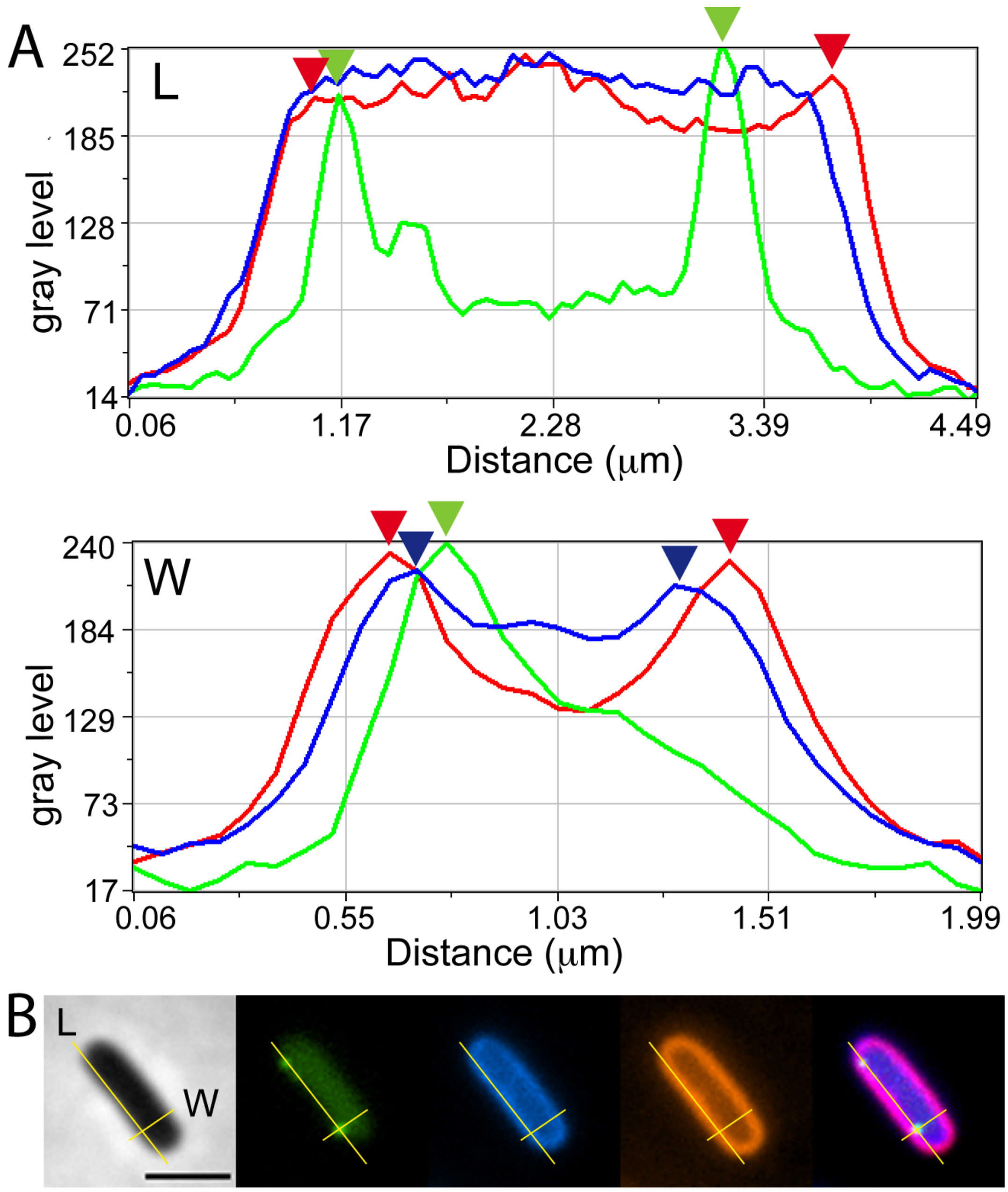

Figure S2: Linescans of fluorescent signals in an Ndd-treated cell.
(A) Linescan analysis of fluorescence signals along cell length (L, top panel) and cell width (W, middle panel). Linescan of fluorescence intensities (Y-axis, in Gray Level units) of cell membrane (red); DNA (blue) and YFP-ParB (green) are shown along the two cell axis (X-axis in μm). Red arrowheads indicate the cell boundaries, blue arrowheads the maximum intensity of the DNA stain and green arrowheads show the positions of YFP-ParB foci. (B) The bottom panel shows micrographs of the cell scanned in the panels above with the two linescans used (from left to right: phase contrast; YFP-ParB; DNA; membrane; overlay YFP-ParB/DNA/membrane. Scale bar is 2μm.

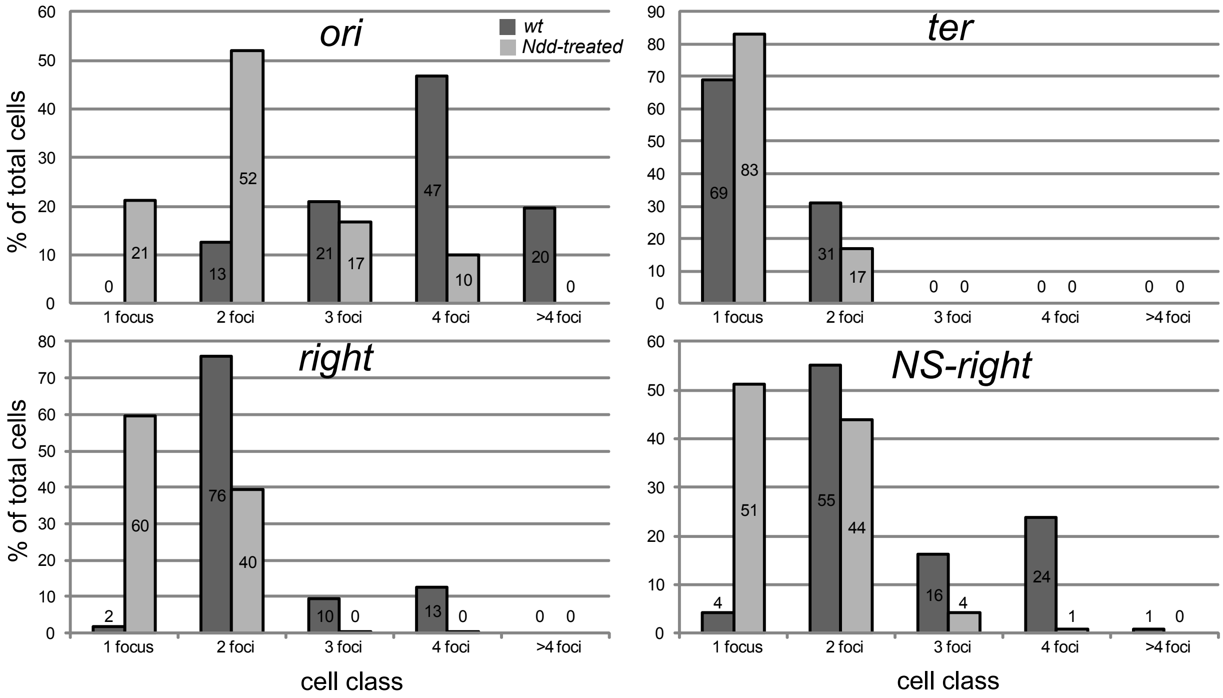

Figure S3: Number of foci per cell in wt and Ndd-treated cells.
Histograms show the distribution of cells in the different cell classes according to their number of foci (X-axis) for the indicated loci. Values are given as percentages of total cells and shown on the histograms (Y-axis). Dark grey: Ndd-untreated cells; Pale grey: Ndd-treated cells.

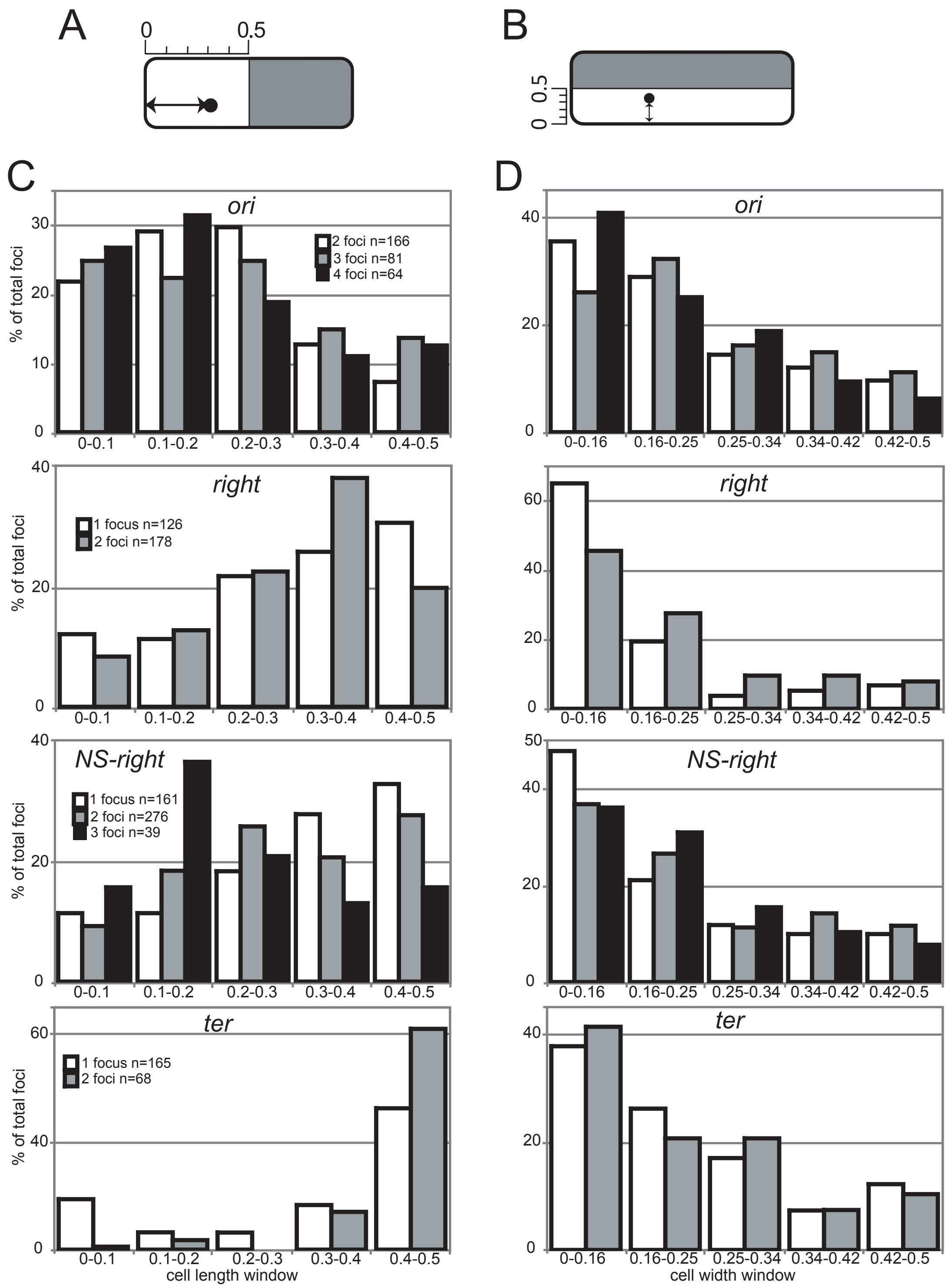

Figure S4: Positionning of foci in Ndd-treated cells.
(A) Drawing showing the measurement of the positions of foci along cell length. The distances of foci centres to their closest cell pole were measured. (B) Drawing showing the measurement of the apparent positions of foci along the cell diameter. The distances of foci centres to their closest membrane along the cell diameter were measured. (C) Distributions of foci of the indicated loci along cell length in the different cell classes. Distributions are plotted as the percentage of total foci in each cell class (Y-axis). The sample size of the cell classes is given on each graph. Foci positions were sampled into five cell length windows (from 0 at the cell periphery to 0.5 at the cell centre, X-axis). (D) Distributions of foci along the cell diameter for the indicated loci in the different cell classes. Distributions are plotted as the percentages of total foci in each cell class (Y-axis). The sample size of the cell classes is given on each graph. Foci positions were sampled into five windows corresponding to cell slices of equivalent areas (from 0 at the cell periphery to 0.5 at the cell centre, X-axis)

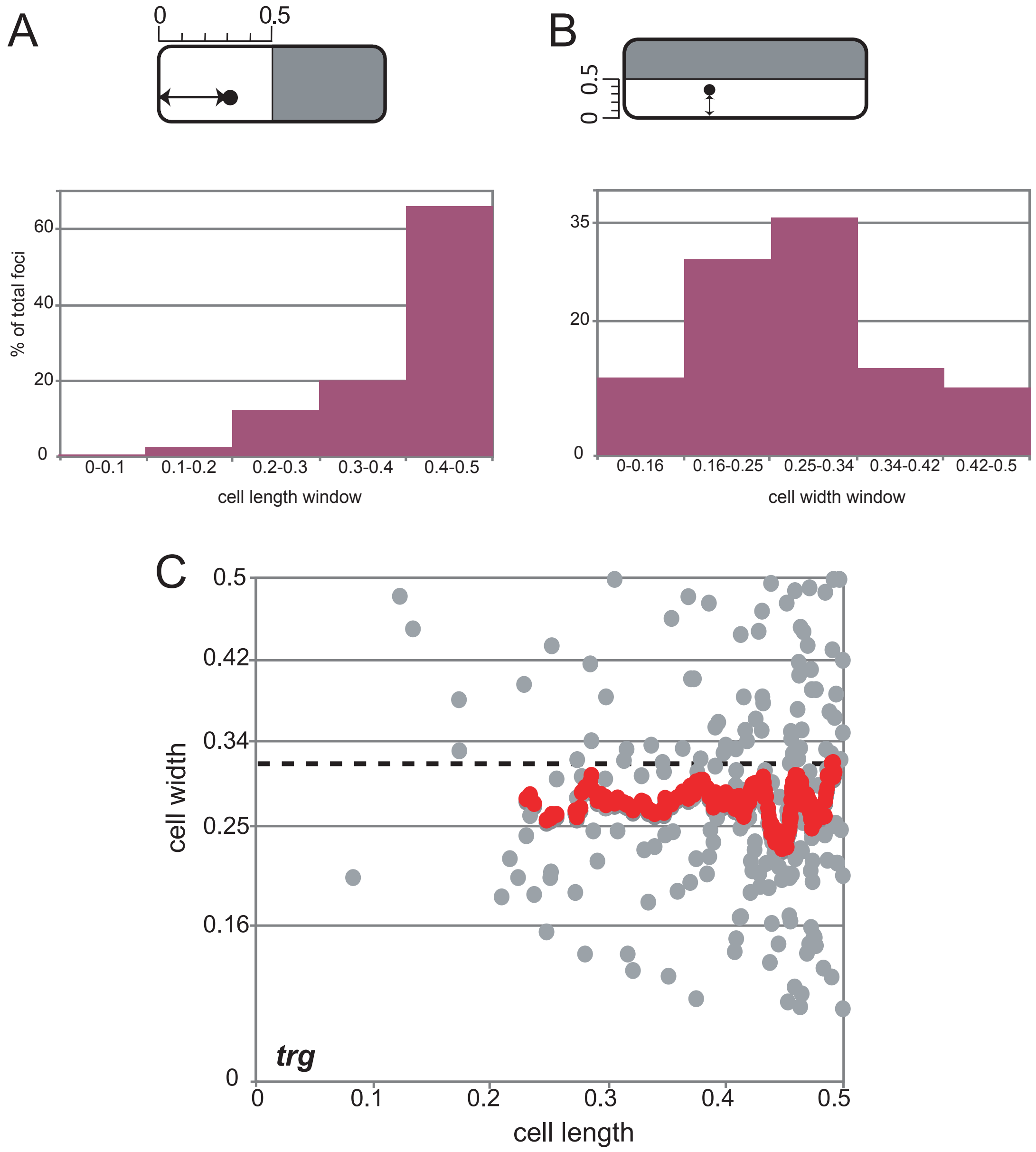

Figure S5: Positionning of the *trg* locus in wt cells.
The *trg* locus is located at 1490 kb. Cells were grown in M9 medium suplemented with casamino acids and glucose at 30°C and observed as described in Methods. Only cells harbouring a single fluorescent focus were considered. (A) Distributions of foci along cell length. Foci positions were sampled into five cell length windows (from 0 at the cell periphery to 0.5 at the cell centre, X-axis). (B) Distributions of foci along the cell diameter. Foci positions were sampled into five windows corresponding to cell slices of equivalent areas (from 0 at the cell periphery to 0.5 at the cell centre, X-axis). (C) Foci positions along the cell diameter (Y-axis) as a function of their cell length positioning (X-axis). The grey dots are individual foci. The red dots are a sliding mean of twenty foci (with a step of one focus). The dotted lines show the mean positioning calculated from the 90% central model.
